# Fertilization and neonatal outcomes after early rescue intracytoplasmic sperm injection: a retrospective analysis of 16,769 patients

**DOI:** 10.1007/s00404-022-06445-z

**Published:** 2022-04-05

**Authors:** Jun Zeng, Zhongyuan Yao, Yeqing Zhang, Fen Tian, Tingting Liao, Lingqian Wu, Yanping Li

**Affiliations:** 1 Reproductive Medicine Center, Xiangya Hospital, Central South University, Changsha, Hunan 410000 People’s Republic of China; 2 Clinical Research Center for Women’s Reproductive Health in Hunan Province, Hunan, 410000 People’s Republic of China; 3 Center for Medical Genetics and Hunan Key Laboratory of Medical Genetics, School of Life Sciences, Central South University, Changsha, Hunan 410000 People’s Republic of China

**Keywords:** Early-rescue ICSI, IVF, Total fertilization failure, Neonatal outcome, Congenital birth defects

## Abstract

**Purpose:**

To evaluate the efficacy and safety of short-term insemination and early-rescue intracytoplasmic sperm injection (ICSI), an approach that rescued oocytes with unclear second polar body 6 h after initial insemination by ICSI (early R-ICSI) to avoid total or near-total fertilization failure in conventional in vitro fertilization (IVF).

**Methods:**

We performed a retrospective study in 16,769 patients (short-term IVF, *n* = 12,094; ICSI, *n* = 3452; early R-ICSI, *n* = 1223) who received IVF/ICSI treatment in our hospital from January 2009 to October 2018. Fertilization and clinical outcomes were compared among those three groups.

**Results:**

When considering the R-ICSI embryos in the early R-ICSI group independently, the rates of fertilization and day-3 cleaved embryos in 2PN oocytes were comparable, the rates of fertilization (2PN) and high-quality embryos were lower, whereas the multi-PN fertilization rate (3.27%) was significantly higher than the ICSI group (1.26%). The difference of clinical pregnancy rate between the part of transferred R-ICSI embryos (40.81%) and the ICSI group (44.73%) remained nonsignificant. Furthermore, the rate of congenital birth defects in the early R-ICSI group (0.99%) was not significantly different from those in the short-term IVF (0.76%) and ICSI groups (1.07%).

**Conclusion:**

Despite the multi-PN fertilization rate, our study highlights early R-ICSI as a safe and effective alternative in assisted reproduction to decrease complete IVF fertilization failure and reduce ICSI utilization. Additional large amount and long-term follow-up studies are needed to further validate the use of early R-ICSI.

## Introduction

In conventional in vitro fertilization (IVF), the possibility of total fertilization failure (TFF) or near-total fertilization failure (NFF) remains inevitable, although the technology of IVF-embryo transfer (ET) has improved currently. TFF or NFF leaves the embryologists with limited alternatives. The first measure is to give up on the present cycle and to offer intracytoplasmic sperm injection (ICSI) directly in a subsequent cycle which, however, increases financial costs associated with infertility treatment. The second alternative is to provide rescue ICSI (R-ICSI) in the current cycle.

R-ICSI in fertilization failure cases was described as re-insemination of the unfertilized oocytes by ICSI when they were nearly 1-day-old. However, it yields poor fertilization results (24–48%) and pregnancy rates (6–20%) [1, 2]. Poor results were reported because of oocyte aging [3] and asynchronization between endometrial growth and embryo development [4]. Frozen embryo transfer seemed to improve with pregnancy rates (31–41%) and implantation rate (11–27%) [5], but it made no difference with fertilization. Subsequently, R-ICSI was performed earlier in an attempt to improve pregnancy rates since the 1990s [6–8]. Early R-ICSI was provided to those oocytes with unclear release of the second polar body 6 h after initial insemination [9], since the second polar body was reportedly released in nearly 90% of fertilized oocytes by 6 h [10, 11].

Fertilization observation time is the important stage for combining spermatozoa and oocytes and the release of the second polar body. Additionally, the early mechanical desorption of cumulus cells may affect the embryo development potential. But in consideration of the removal of cumulus cells in ICSI, it is even earlier than that in early R-ICSI. As an increasing use of ICSI and around two-thirds of fresh cycles in Europe [12] and the United States (according to SART.org 2017), there is no proof that the removal of cumulus cells in ICSI influenced the development of oocytes and embryos.

Combining short co-incubation and early R-ICSI could improve the clinical outcomes of patients with failed IVF, but this strategy has not been evaluated adequately. In this study, we aimed to retrospectively investigate the effect of early cumulus-cell removal on embryo development potential and clinical outcomes, and to discuss the safety and efficacy of short insemination combined with early R-ICSI.

## Methods

### Patients

In this study, patients who underwent IVF, ICSI, and R-ICSI treatment in Xiangya Hospital of Central South University from January 2009 to October 2018 were retrospectively analyzed. To identify the broad range of patients typically encountered in clinical practice and exclude the influence of previous ovarian hyperstimulation, patients who received their first cycle of IVF/ICSI treatment were included. The study comprised 13,317 patients who underwent short insemination and early cumulus-cell removal for fertilization observation. Those who received early R-ICSI treatment (*n* = 1223) were classified as the early R-ICSI group, whereas those just received short-term IVF (*n* = 12,094) were classified as the short-term IVF group. 3452 patients were recommended to undergo ICSI treatment directly because of severe oligospermia. This study was approved by the Institutional Review Board from the Ethics Committee of Reproductive Medicine Center, Xiangya Hospital, Central South University. Informed consents were unnecessary because the research was based on non-identifiable records, as approved by the ethics committee.

### Clinical procedures and oocyte retrieval

Our center has been following a consistent treatment plan and procedures for ovulation induction and embryo culture. Couples in this study were all given stimulated procedure for ovulation induction.

When two or more follicles reached a mean diameter of 18 mm, the human chorionic gonadotropin was administered. Transvaginal and ultrasound-guided follicular aspiration was performed 35–36 h after HCG injection. Furthermore, cumulus–corona–oocyte complexes were cultured for 2–3 h after oocyte retrieval.

### Sperm preparation

Semen samples were collected by masturbation after 2–7 days of sexual abstinence during oocyte retrieval. The ejaculates were maintained for at least 30 min at 37 °C for liquefaction. We then assessed the semen samples according to published criteria for sperm density, motility, and morphology (World Health Organization [WHO], 1999 and WHO 2010 manual). Sperm density ≥ 15 × 10^6^/ml and progressive sperm ≥ 32% were regarded as normal. Liquefied ejaculates were prepared by conventional discontinuous density gradient centrifugation and swim-up procedures in accordance with the WHO guideline. The processed sperm remained incubated for the next insemination.

### In vitro insemination and short co-incubation

IVF is generally performed 39–40 h after HCG injection, allowing to occur naturally. Each oocyte is incubated with approximately 20,000 sperm cells. Short co-incubation was adopted, and the cumulus granule cells were peeled off [13] 4–6 h after fertilization.

Patients received ICSI directly when sperm density ≤ 5 × 10^5^/ml after the process, when sperms were surgically retrieved, or when the patient had previous fertility failure in other centers. Patients treated with ICSI had their metaphase II (MII) oocytes microinjected with sperms 3–4 h after oocyte retrieval. Sperms with normal morphology were selected, immobilized, and injected into the oocyte cytoplasm.

### Fertilization evaluation and early R-ICSI

After 4–6 h of co-incubation, fertilization was observed under a microscope (100 × –200 ×). If the second polar body was extruded, the oocyte was considered fertilized. In patients with a missing second polar body in any of the retrieved oocytes or with a low fertilization rate (< 30%), the MII oocytes (number ≥ 1) would be rescued to undergo the same ICSI method.

### Embryo culture and transfer

All of the embryos from short-term IVF, ICSI, and early R-ICSI cycles were checked on the morning of days 1, 2, and 3 after oocyte retrieval. Day-3 embryos can be classified into four levels as follows according to their quality: Level I (cells > 6, uniform cell size, < 5% cell fragments); Level II (cells > 6, slightly nonuniform cell size, < 20% cell fragments); Level III (cells between 4 and 6 or 20–50% cell fragments); and Level IV (> 50% cell fragments or no cell division in 24 h). Levels I and II embryos were considered as high-quality embryos.

On day 3, 2–3 best-quality embryos were chosen for transfer. Transferring three embryos are only considered for the following reasons: advanced age, repeated failure, and without contraindications. For each level, embryos formed from IVF were preferred. Those patients with ovarian stimulation syndrome, endometrial factors, or personal reasons got embryos frozen and not transferred. The luteal-phase support was sustained with natural progesterone from the oocyte retrieval day. The remaining embryos were used to culture blastocysts, freeze, or ruin.

### Outcome measures

Clinical pregnancy was confirmed if one or more gestational sacs were detected by transvaginal ultrasound at 28 days after the ET. The implantation rate is the number of observed gestational sacs divided by the number of transferred embryos. We calculated the clinical pregnancy rate by dividing the number of clinical pregnancies by the number of patients. An intrauterine pregnancy that fails to reach 28 weeks of gestation indicates miscarriage. Live birth was defined as the delivery of a live infant after 24 weeks of gestation. The neonatal outcome data were obtained by telephone interview of the parents after delivery. Information on gestational weeks, sex, birth weight, and congenital birth defects was determined using a questionnaire.

### Statistical analysis

Data were analyzed using the SPSS 25.0 for Windows. The baseline characteristics were expressed as the mean ± standard deviation (SD) and analyzed using one-way analysis of variance and Duncan’s multiple-range tests. Categorical variables were expressed in percentage and compared using chi-square test. Moreover, *p* < 0.05 indicated statistical significance.

## Results

In brief, 16,769 ovarian stimulation cycles (short-term IVF, *n* = 12,094; ICSI, *n* = 3452; early R-ICSI, *n* = 1223) were analyzed in this study. Short-term insemination was performed on 13,317 patients. During the short insemination treatment, the incidence of NFF was 6.49% (864/13,317), whereas that of TFF was 4.21% (561/13,317). During the procedures of short co-incubation, 1,223 patients presented a less than 30% fertilization rate and underwent early R-ICSI treatment (early R-ICSI group), and 202 patients did not get rescue as oocytes immature or too few. Then, the incidence of NFF and TFF was reduced to 2.27% (302/13,317) and 1.08% (144/13,317), respectively. In addition, the NFF and TFF were 2.06% (71/3,452) and 2.67% (92/3,452) in the ICSI group.

The baseline characteristics of the ICSI and early R-ICSI groups are shown in Table 1. The three groups had no significant differences in terms of the patients’ age, body mass index, and basal FSH and luteinizing hormone levels and stimulation time and dosage. The early R-ICSI group had a significantly longer infertility duration than the short-term IVF group. Additionally, the proportions of primary infertility among the short-term IVF, ICSI, and early R-ICSI groups were 38.81, 58.11, and 50.45%, respectively, which were all significantly lower (*P* < 0.001) in the short-term IVF group than in the ICSI and early R-ICSI groups. The early R-ICSI group had significantly lower E2 level on HCG day and mean number of oocytes retrieved than the short-term IVF and ICSI group.

**Table 1:** Baseline characteristics of the short-term IVF, ICSI and early-rescue ICSI groups

Parameter	Short-term IVF group	ICSI group	Early-rescue ICSI group
No. of patients	12,094	3452	1223
Male age (years)	34.02 ± 5.89	34.16 ± 6.48	33.82 ± 6.15
Female age(years)	31.55 ± 5.19	31.18 ± 5.53	31.26 ± 5.22
BMI of female (kg/m^2^)	21.85 ± 2.92	21.76 ± 2.89	21.79 ± 2.90
Duration of infertility (years)	4.64 ± 3.54^a^	4.99 ± 3.88	4.86 ± 3.56 ^b^
Infertility type			
Primary infertility (%)	4694 (38.81)^a^	2006 (58.11)^b^	617 (50.45)^c^
Secondary infertility (%)	7400 (61.19)^a^	1446 (41.89)^b^	606 (49.55)^c^
Unexplained infertility	213 (1.76)	88 (2.55)	20 (1.64)
Basal FSH (IU/L)	7.01 ± 3.22	6.98 ± 2.64	7.01 ± 2.73
Basal LH (IU/L)	5.76 ± 4.47	5.58 ± 5.44	5.66 ± 3.93
Basal E2 (pmol/L)	44.39 ± 72.77	42.27 ± 78.54	44.47 ± 52.07
Duration of stimulation (days)	10.72 ± 3.05	10.60 ± 2.88	10.75 ± 3.32
Dosage of gonadotrophin (Gn)	2043 ± 856	2013 ± 840	2073 ± 1091
LH on HCG day (IU/L)	2.43 ± 3.66	2.40 ± 3.42	2.42 ± 3.90
E2 on HCG day (pmol/L)	3514 ± 2453^a^	3385 ± 2378^b^	3126 ± 2182^c^
P on HCG day (nmol/L)	0.99 ± 3.06	0.98 ± 2.13	1.03 ± 3.57
Endometrial thickness on ET day (mm)	10.72 ± 2.24	10.81 ± 2.18	10.75 ± 2.16
Mean (± SD) of oocytes retrieved	11.50 ± 6.40^a^	11.24 ± 5.19^b^	10.71 ± 6.16^c^

Values are presented as mean ± SD, unless otherwise noted. In each row, values with different superscript letters differ significantly (*P* < .05)

Fertilization outcomes of the embryos that underwent R-ICSI (R-ICSI embryos) in the early R-ICSI group are shown independently in Table 2. When considering the R-ICSI embryos, the fertilization rate was higher than those in the short-term IVF and ICSI group; the rates of fertilization (2PN) and high-quality embryos were lower than the ICSI group but higher than the short-term IVF group, whereas the multi-PN fertilization rate was significantly higher than the ICSI group but lower than the short-term IVF group.

**Table 2:** Laboratory data of the short-term IVF group, ICSI group and R-ICSI embryos in the early-rescue ICSI group

Parameter	Short-term IVF group	ICSI group	R-ICSI embryos
No. of cycles	12,094	3452	1223
No. of oocytes retrieved/MII	139,135	30,854	7562
Fertilization rate (%)	107,092 (76.97)^a^	23,781 (77.08)^a^	5912 (78.18)^b^
Fertilization rate (2PN) (%)	93,416 (67.14)^a^	22,759 (73.76)^b^	5477 (72.43)^c^
Multi-PN fertilization rate (%)	8340 (5.99)^a^	389 (1.26)^b^	247 (3.27)^c^
Day-3 cleaved embryos/2PN oocytes (%)	90,714 (97.21)^a^	22,025 (96.77)^b^	5294 (96.66)
High-quality embryo rate (%)	57,975 (58.71)^a^	13,234 (56.57)^b^	2869 (50.64)^c^

In each row, values with different superscript letters differ significantly (*P* < .05)

In the early-rescue ICSI group, patients could be divided into three parts according to the origin of embryos transferred: part of transferred short-term insemination embryos, part of transferred R-ICSI embryos, and part of transferred short-term insemination embryos and R-ICSI embryos together. To analyze the safety and efficacy of rescue ICSI, we compared the part of transferred R-ICSI embryos independently (Table 3). The number of ET cycle and the number of transferred embryos had no significant difference. Despite the significantly lower implantation rate, the difference of clinical pregnancy rate and miscarriage rate among the three groups remained nonsignificant. Moreover, the causes of abortion were not significantly different in three groups. No significant differences were also observed in the live birth rate in the two groups, but the number of singletons in the early R-ICSI group was significantly higher than that in the Short-term IVF and ICSI group.

**Table 3:** Clinical outcomes of the short-term IVF group, ICSI group and the part of transferred R-ICSI embryos in the early-rescue ICSI group

Parameter	Short-term IVF group	ICSI group	Part of transferred R-ICSI embryos
No. of ET (cycle)	8725	2361	571
No. of embryos transferred	16,722	4488	1077
Mean(± SD) of embryos transferred	1.92 ± 0.32	1.90 ± 0.37	1.89 ± 0.35
Clinical pregnancy rate (%)	4064 (46.58)^a^	1056 (44.73)	233 (40.81)^b^
Embryo implantation rate (%)	5601 (33.49)^a^	1428 (31.82)^b^	296 (27.48)^c^
Miscarriage rate (%)	631 (15.53)	165 (15.63)	30 (12.88)
Early abortion (< 12 weeks, %)	400 (63.39)	102 (61.82)	14 (46.67)
Embryo cessation	389 (97.25)	102 (1.00)	14 (1.00)
Late abortion (≥ 12 weeks, %)	231 (36.61)	63 (38.18)	16 (53.33)
Embryo cessation	131 (56.71)	41 (65.08)	9 (56.25)
Placental factors	39 (16.88)	11 (17.46)	5 (31.25)
Infant deformity	22 (9.52)	7 (11.11)	1 (6.25)
Others	39 (16.88)^a^	4 (6.35)^b^	1 (6.25)
Live birth rate (%)	3433 (39.35)	890 (37.70)	203 (35.55)
Singletons (% per live delivery)	2292 (50.02)^a^	662 (59.05)^b^	167 (69.58)^c^
Twins (% per live delivery)	1133 (49.45)^a^	225 (40.14)^b^	35 (29.17)^c^
Triplets (% per live delivery)	8 (0.52)	3 (0.80)	1 (1.25)

In each row, values with different superscript letters differ significantly (*P* < .05)

Table 4 shows the neonatal outcomes of singleton and multiple gestations for the ICSI group and the part of transferred R-ICSI embryos in the early R-ICSI group. For singleton deliveries in the two groups, the delivery method, mean gestational age, preterm deliveries, and very preterm deliveries of the early R-ICSI group were not significantly different. A total of 256 babies (133 male and 123 female) were born from the early R-ICSI embryos. Their mean birth weight was 3,200 ± 575 g, comparable with the singleton deliveries from the ICSI group (3,220 ± 566 g, *P* = 0.684). Furthermore, the number of babies grouped by the birth weight was not significantly different. For multiple gestations, regarding the delivery method of neonates, the early R-ICSI group was more likely to undergo cesarean sections. Compared with the ICSI group, the early R-ICSI group had a significantly longer mean gestational age range and had a significantly heavier mean birth weight. Meanwhile, no significant differences were identified in sex ratio and birth defects.

**Table 4:** Neonatal outcomes of the short-term IVF, ICSI group and the part of transferred R-ICSI embryos in early-rescue ICSI group

	Singleton gestation			Multiple gestation		
Parameter	Short-term IVF group	ICSI group	Part of transferred R-ICSI embryos	Short-term IVF group	ICSI group	Part of transferred R-ICSI embryos
No. of live birth	2292	662	167	2290	459	73
No. of vaginal deliveries	764 (33.33)	239 (36.10)	54 (32.34)	178 (7.77)^a^	34 (7.41)^a^	0^b^
No. of cesarean sections	1528 (66.67)	423 (63.90)	113 (67.66)	2112 (92.23)^a^	425 (92.59)^a^	73 (1.00)^b^
Mean gestational age (weeks)	38.51 ± 2.35	38.64 ± 1.85	38.66 ± 2.19	36.22 ± 2.24	36.37 ± 2.08^a^	36.79 ± 1.51^b^
Preterm deliveries (< 37 weeks)	151 (6.59)	44 (6.65)	12 (7.19)	924 (40.35)	174 (37.91)	31 (42.47)
Very preterm deliveries (< 32 weeks)	21 (0.92)	7 (1.06)	3 (1.80)	101 (4.41)	12 (2.61)	0
Mean birth weight (g)	3235 ± 540	3220 ± 566	3200 ± 575	2418 ± 554	2457 ± 493^a^	2565 ± 378^b^
Birth weight < 1500 g	15 (0.66)	6 (0.91)	3 (1.80)	85 (3.75)	12 (2.63)	0
Birth weight 1500–2499 g	114 (4.99)	36 (5.44)	9 (5.39)	1004 (44.27)	204 (44.64)	26 (35.62)
Birth weight 2500–3999 g	1997(87.36)	583 (88.07)	144 (86.23)	1174 (51.76)^a^	242 (52.72)	47 (64.38)^b^
Birth weight ≥ 4000 g	160 (7.00)	37 (5.59)	11 (6.59)	5(0.22)	1 (0.22)	0
Sex ratio, male/female	1.16, 1229/1063	1.14, 352/310	0.99, 83/84	1.18, 1239/1051	1.09, 239/220	1.09, 38/35
Total birth defects/total live birth (%)	24 (1.05)	8 (1.21)	3 (1.80)	11 (0.48)	4 (0.87)	1 (1.37)

In each row, values with different superscript letters differ significantly (*P* < .05)

Table 5 shows the incidence and type of congenital birth defects. Of the 6109 live births in all three groups, 0.83% (51/6,109) newborns showed birth malformations. Despite those malformations of unclear reasons, cardiovascular defects accounted for the majority of birth defects, followed by musculoskeletal and urogenital defects. But in our study, congenital birth defects obtained by parental reports after delivery showed no significant differences in the outcomes of newborns delivered after early R-ICSI cycles (0.99%) vs. short-term IVF (0.76%) or ICSI cycles (1.07%).

**Table 5:** Stillbirths and neonatal malformations in short-term IVF, ICSI, and the part of transferred R-ICSI embryos in the early-rescue ICSI groups

Parameter	Short-term IVF group (%)	ICSI group (%)	Part of transferred R-ICSI embryos (%)
Total live births and stillbirths	4609	1127	406
Stillbirths	27 (0.59)	6 (0.53)	0
Live births	4582	1121	406
Malformations types			
Chromosomal	1 (0.02)	0	2 (0.49)
Cardiovascular	13 (0.28)	4 (0.36)	0
Musculoskeletal	3 (0.07)	0	0
Urogenital	0	3 (0.27)	0
Nervous	2 (0.04)	0	0
Respiratory	0	1 (0.09)	0
Digestive system	0	0	1 (0.25)
Other congenital malformations	0	1 (0.09)	0
Neonatal deaths of unclear reasons	17 (0.37)	3 (0.27)	1 (0.25)
Total birth defects/total live birth	35 (0.76)	12 (1.07)	4 (0.99)

## Discussion

In conventional IVF cycles, TFF or NFF occurs in 5–20% [14–18], and it remains unavoidable despite the development of assisted reproductive technology. For the past decade, R-ICSI has been used as one of the saving methods. Based on the current available literature, considerably higher clinical pregnancy rates, which range from 43 to 61% [13, 19–21], were observed in early R-ICSI than in late R-ICSI [3, 4, 22, 23]. Late R-ICSI was not selected because it can lead to poor clinical pregnancy outcome resulting from oocyte aging [3] and asynchronized endometrium [4].

Primary infertility [24] or longer infertility duration [25] is an important risk factor for TFF; for patients with unexplained infertility, the incidence of TFF could be as high as 17.6–25% [26]. However, the exact reason of unexplained infertility remains unknown and may be associated with some potential causes, including fertilization defect, endocrine disorders, immunological defects, genetic and reproductive physiology, and zona pellucida hardening or meiotic errors [24]. In the current study, the proportion of primary infertility and infertility period in the early R-ICSI group was significantly higher than that in the short-term IVF group. It suggested that patients with primary infertility and longer infertility duration should routinely undergo short insemination combined with early cumulus-cell removal and receive early R-ICSI in their reproductive center if necessary.

Note that this is a source of great controversy as early-rescue ICSI presents early mechanical desorption of cumulus cells and shows increased polyspermy rate in previous studies. During the conventional IVF procedure, the co-culture of oocytes and cumulus cells for 18–20 h was considered to improve embryo morphology and blastocyst formation [27]. Cumulus cells provide oocytes with a series of factors, including glycosaminoglycan, steroid hormones, and nutrients, which play important roles for oocyte nuclear and cytoplasmic maturation, fertilization, and development [28]. In contrast, other studies [11] showed a significantly higher fertilization rate or available embryo rate in the short-term IVF group than in the traditional IVF group, probably because the removal of cumulus cells reduces the levels of toxic metabolites produced by cumulus cells and sperms; these toxic metabolites have detrimental effects on the embryo developmental potential along with the extension of incubation time [29, 30]. Besides, a prospective randomized sibling-oocyte study [31] showed the 3 h group, when compared with the 20 h group, had higher rates of optimal quality embryos and polyspermy, but no differences in their rates of normal fertilization, pregnancy, and live birth. It should be clearly noted that the removal of cumulus cells in ICSI procedure is presented 3–4 h after oocyte retrieval, earlier than that in R-ICSI group. But there is no proof that the removal of cumulus cells influenced the development of oocytes and embryos after decades of ICSI [26, 32].

However, the effects of early removal of the cumulus cells on polyspermy still remain controversial according to previous reports. In the study of Lundqvist et al., short-term IVF obtained a lower normal fertilization rate than the conventional IVF [33]. Meanwhile, the oocytes are more vulnerable because of the presence of active spindles and microtubules at an early time after insemination, and more repeated aspirations may be detrimental to the integrity of oocyte structure, reducing its defense against polyspermy [13]. Moreover, the experience of the embryologist who observes the polar body to evaluate the nonfertility and performs the R-ICSI is also an important influencing factor. These factors would influence the normal fertilization process and increase the poly-spermic fertilization. However, short-term insemination and early R-ICSI frequently do not increase the polyspermy rate [13, 17, 30, 34]. Our research showed that the polyspermy rate in the short-term IVF and the early R-ICSI groups was significantly higher than that in the ICSI group. Thus, early R-ICSI helped patients avoid complete IVF fertilization failure, but at the same time, it may bring a certain degree of excessive treatment to oocytes with undefined fertilization status. New approaches, such as spindle imaging using polarization microscopy combined with rescue measures [35], could be applied to effectively prevent fertilization failure and decrease the polyspermy rate.

Moreover, unfertilized oocytes resulting from failure of the sperms to travel through the zona pellucida, such as in the case of oligospermia, asthenospermia, or teratozoospermia, can be saved through early R-ICSI. The possible negative effect of isolated teratozoospermia on IVF is controversial. The rates of fertilization, implantation, pregnancy, and lower-quality embryos are abnormally low in the IVF cycles of patients with teratozoospermia [36, 37]. However, a study by Keegan et al. found no improvement in the IVF outcomes when ICSI was used to treat couples with isolated teratozoospermia; of note, sibling oocytes were not used in that study [38]. Conversely, Fan et al. [39] found similar results using sibling oocytes. Younes et al. [37] found that when normal sperm morphology ≤ 4%, ICSI over IVF can obtain a higher number of cleavage-stage and day-5 embryos as well as better-quality blastocysts. In our study, R-ICSI embryos, which remained unfertilized after short-term IVF, had a comparable fertilization rate (78.18%), and day-3 cleaved embryos/2PN oocytes obtained a high cleavage rate (96.66%) in the ICSI group. To our knowledge, we are the first to analyze all embryos that received early R-ICSI independently.

When considering R-ICSI embryos independently, the sperms were microinjected into oocytes 4–6 h later compared with those in the ICSI group. Our study showed that the R-ICSI embryos had lower rates of normal fertility, high-quality embryos, and implantation than the ICSI group, probably because of the oocyte aging and subsequent embryonic development. However, these impairments were insufficient to change the outcomes, considering that the difference of clinical pregnancy rate and miscarriage rate between the ICSI group and the part of transferred R-ICSI embryos remained nonsignificant. Early R-ICSI requires standardized protocols that can answer the following questions: What sign and fertilization observation time are appropriate to check for fertilization? For the second polar body at more or less 6 h, when is the right time to perform ICSI that can achieve a better outcome?

Data on newborns delivered after employing R-ICSI embryos are few, most especially the outcomes associated with early R-ICSI. In our study, no significant differences were observed in miscarriage rate and live birth rate among the three groups, indicating that short-term insemination had no influence on the outcomes of IVF-ET. Additionally, the pregnancy outcomes after early R-ICSI were acceptable, suggesting that this technique was a safe and effective alternative method to prevent fertilization failure or a lower fertilization rate during the conventional IVF treatment. Cumulus cells may have both beneficial and adverse effects on oocytes; further studies are needed to confirm the exact nature.

Furthermore, data on neonatal outcomes after R-ICSI cycles are extremely limited. In our study, total birth defects showed no significant differences among the three groups, and were mainly consisted of cardiovascular, musculoskeletal, and urogenital defects. A large number of studies have followed up on the growth and development of ART-conceived human offspring. Multicenter epidemiological studies have shown that the incidence of neonatal defects, cardiovascular, musculoskeletal, and urogenital malformations accounting for the majority, in ART-conceived offspring is significantly increased compared with that of naturally conceived newborns [40–42]. The process of in vitro embryo culture, known as embryogenesis, is one of the two critical periods where epigenetic reprogramming occurs during mammalian development [43]. Epidemiological studies have described that any defects during epigenetic reprogramming, including the imprinting process, may increase the risk of genetic and epigenetic disorders [44, 45]. Mouse models have been used to study and observe embryonic abnormalities and offspring diseases [46, 47], but the precise molecular mechanisms underlying these malformations remain unclear. It is a limit that congenital birth defects were based on telephone interviews; besides, the birth defects may need further genetic testing and phenotypic analysis. Additional large amount and long-term follow-up studies are needed to further validate the safety and efficacy of early R-ICSI.

Of note, ICSI has higher costs, procedure times [32], chances of damaging the oocyte [48], and proteomic alterations [49], as well as possibly higher rates of fetal anomalies [50]. The first 15 years of ART activity in Europe (1997–2011) showed an increasing proportion of ICSI and it exceeded the use of IVF from 2002 onwards. ICSI represented around double of IVF since 2008 [12]. According to SART.org 2017, ICSI was applied to 75.4% of infertile patients in the United States, accounting for 27.9% in our study. Given that the clinical and neonatal outcomes after the R-ICSI cycles are comparable with those after the ICSI cycles, we recommend short-term insemination with early R-ICSI as an alternative method of ICSI in some cases.

In conclusion, our results clearly show that short co-incubation combined with early cumulus-cell removal could achieve satisfying clinical and neonatal outcomes compared with ICSI. Therefore, short-term insemination can be widely used for patients, especially those with primary longer infertility, and early R-ICSI can be employed in early-stage post TFF or NFF.

## Data Availability

All data are available in this paper.

## Abbreviations

IVF: In vitro fertilization
ICSI: Intracytoplasmic sperm injection
early R-ICSI: Early-rescue ICSI
TFF: Total fertilization failure
NFF: Near-total fertilization failure
ET: Embryo transfer
